# Plant-Dominant Low-Protein Diets: A Promising Dietary Strategy for Mitigating Disease Progression in People with Chronic Kidney Disease—A Comprehensive Review

**DOI:** 10.3390/nu17040643

**Published:** 2025-02-11

**Authors:** Jun-Ya Kaimori, Yusuke Sakaguchi, Tatsufumi Oka, Yoshitaka Isaka

**Affiliations:** 1 Department of Health and Nutrition, Otemae University, 2-1-88 Otemae, Chuo-ku, Osaka 540-0008, Japan; 2 Department of Nephrology, Osaka University Graduate School of Medicine, Suita 565-0871, Japan; sakaguchi@kid.med.osaka-u.ac.jp (Y.S.); oka@kid.med.osaka-u.ac.jp (T.O.); isaka@kid.med.osaka-u.ac.jp (Y.I.)

**Keywords:** chronic kidney disease (CKD), plant-dominant low-protein diets (PLADO), low-protein diets, plant-based diets

## Abstract

Chronic kidney disease (CKD) is a global health crisis affecting over 10% of the population, with mortality rates increasing significantly. Current management strategies, including expensive medications and renal replacement therapies, highlight the need for cost-effective, conservative approaches. This review examines the evidence for plant-dominant low-protein diets (PLADO) in managing non-dialysis-dependent CKD. Existing guidelines for protein restriction in CKD vary considerably, with inconsistencies and a lack of personalization noted in the KDOQI and KDIGO recommendations. While traditional low-protein diet trials show limited success due to poor adherence and marginal benefits, PLADO offers a potentially more sustainable alternative. PLADO’s advantages include improved nutrient density, reduced dietary acid load, anti-inflammatory effects, and beneficial modulation of the gut microbiome, potentially reducing uremic toxins and improving cardiovascular health. However, challenges remain, including adherence issues, potential nutrient deficiencies, and potassium management. Although observational studies show promise, further large-scale randomized controlled trials are necessary to validate PLADO’s efficacy and establish optimal dietary composition. A personalized, multidisciplinary approach is essential for successful implementation and monitoring to maximize PLADO’s benefits in improving outcomes for individuals with NDD-CKD.

## 1. Introduction

Chronic kidney disease (CKD) constitutes a serious public health issue, affecting over 10% of the global population, or a total of 843.6 million people [1]. CKD has become a major contributor to mortality rates, with a 41.5% increase from 1990 to 2017. Predictions indicate that CKD is likely to become the fifth leading cause of years of life lost worldwide by 2040 [1]. The primary risk factors for CKD are non-communicable diseases, including hypertension [2], diabetes [3], metabolic syndrome, and obesity. CKD encompasses a spectrum of kidney damage and a reduced glomerular filtration rate (GFR), which is associated with the development of anemia; metabolic acidosis, which increases the risk of hyperkalemia; the accumulation of uremic toxins, including phosphorus; and hyperphosphatemia, which leads to elevated blood levels of fibroblast growth factor 23, decreased levels of 1.25-dihydroxy vitamin D3, and secondary hyperparathyroidism [4]. This results in mineral bone diseases and a higher risk of bone fractures and vascular calcifications, leading to cardiovascular events that significantly increase healthcare expenses [5]. People with CKD require expensive drug treatments (such as erythropoiesis-stimulating agents, binders for potassium and phosphate, and calcium mimetics, among others) as well as costly renal replacement therapies (including hemodialysis, peritoneal dialysis, and kidney transplantation) in the later stages of CKD [6]. The rise in the aging population with CKD is expected to create challenges for healthcare systems regarding resource distribution and effective disease management strategies [7]. In response to this situation, researchers have put forth the concept of “conservative and preservative care” for people with CKD. This idea focuses on delivering, as follows: (1) proactive and thorough medical care for advanced CKD through non-dialytic approaches; and (2) slowing the progression of CKD, with the primary goals being enhancing health-related quality of life, addressing the symptoms and complications of kidney failure without the need for dialysis or transplantation, and maintaining residual kidney function [8,9]. PLADO, a plant-dominant, low-protein diet, is a dietary strategy employed within this framework. It involves restricting overall protein intake while emphasizing plant-based protein sources to mitigate CKD progression and enhance the quality of life. Although various therapeutic approaches exist, dietary management can play a crucial role in conservative and preservative care for non-dialysis-dependent (NDD) people with CKD by slowing disease progression and improving patient outcomes in an inexpensive way. In this review article, we explore the current evidence surrounding PLADO in the management of NDD-CKD, examining the mechanisms of action, efficacy, challenges, and future research directions.

## 2. Clinical Practice Guidelines on Low-Protein Diets for People with NDD-CKD

Protein intake is a key component of dietary management in NDD-CKD. Various organizations, including the Kidney Disease Outcomes Quality Initiative (KDOQI), International Society of Renal Nutrition and Metabolism (ISRNM), Kidney Disease: Improving Global Outcomes (KDIGO), and the Japanese Society of Nephrology, have provided guidelines on protein intake for people with CKD. Each offers nuanced recommendations, reflecting differences in clinical priorities and regional health considerations.

### 2.1. KDOQI Clinical Practice Guidelines

The recently updated KDOQI guidelines emphasize individualized patient care, advocating, as follows: (1) a low-protein diet of 0.55–0.60 g/kg body weight/day; or (2) a very-low-protein diet of 0.28–0.43 g/kg of body weight/day with additional keto acid analogs for people with stages G3 to G5 NDD-CKD without diabetes and who have a stable metabolic state. The guidelines recommend a lower dietary protein intake of 0.6–0.8 g/kg of body weight/day for people with stages G3 to G5 NDD-CKD who have diabetes to maintain a stable nutritional status and optimize glycemic control. The guidelines also stress the association of lower-protein diets with delaying the progression to end-stage renal disease (ESRD) [10].

### 2.2. ISRNM Guidelines

The ISRNM guidelines provide a more flexible framework, aligning closely with KDOQI by recommending a protein intake as low as 0.60–0.80 g/kg/day. However, the ISRNM guidelines highlight the importance of essential amino acid supplementation or nitrogen-free analogues in very-low-protein diets to prevent protein–energy wasting, which is a crucial consideration in managing CKD [11].

### 2.3. KDIGO Recommendations

The KDIGO recommendations take a slightly different approach, recommending a dietary protein intake of 0.8 g/kg for people with stages G3 to G5 NDD-CKD but cautioning against very-low-protein diets (0.3–0.4 g/kg) with essential amino acids or ketoacid analogs (up to 0.6 g/kg body weight/d), unless adult people with CKD are willing and able to adhere to such diets under close supervision. The KDIGO recommendations emphasize balancing protein intake to avoid malnutrition while still supporting renal function by reducing nitrogenous waste production [12].

### 2.4. Japanese Society of Nephrology Guidelines

The Japanese guidelines suggest that protein intake should be carefully monitored to avoid excess in CKD stages G1 to G2. In stage G3a, 0.8–1.0 g/kg/day is recommended; in stages G3b and beyond, 0.6–0.8 g/kg/day is suggested. Management in CKD stages G3b and beyond should ideally be undertaken by a multidisciplinary team, including a nephrologist, registered dietitian, and renal dietitian. When protein restriction is intensified, sufficient energy intake is essential. In patients with CKD who have sarcopenia and frailty, protein restriction may be relaxed. This recommended protein intake reflects a cultural context wherein staple diets may naturally include lower protein intake compared with Western diets. It is recommended that the necessary energy consumption and limited protein intake be monitored by a medical team, including a nephrologist and dietitian, as this is anticipated to slow CKD stage advancement [13,14].

This review presents several chronologically ordered CKD protein intake guidelines. A well-known problem in the KDOQI 2020 update’s recommendations on low-protein diets, also acknowledged by the ISRNM, is the lack of consideration for the substantial heterogeneity among CKD patients. The guidelines did not adequately account for variations in individual nutritional needs, comorbidities, and responses to dietary interventions, potentially leading to recommendations that are not universally applicable or beneficial. In essence, the “one-size-fits-all” approach to low-protein diets was criticized for a lack of personalization. The KDOQI guidelines also comment that there is not enough evidence for recommending plant-based protein. While none of the guidelines above explicitly endorse PLADO, inconsistencies exist. The KDOQI and KDIGO guidelines recommend very-low-protein diets, unlike the ISRNM and Japanese Society of Nephrology guidelines, which offer no comment on this approach. Interestingly, the Japanese guidelines recommend a relatively mild low-protein diet without mentioning keto acid analog supplementation.

## 3. Evidence for Favorable Effects of a Low-Protein Diet on Kidney Disease Progression

Discrepancies in these guidelines may arise from variations in the interpretation of clinical studies and diverse patient population data. The KDOQI guidelines have the most aggressive recommendation in terms of protein restriction. The KDOQI guidelines are based solely on the results of randomized controlled trials (RCTs), not those of implementation studies [15]. A recent meta-analysis of low protein intake in NDD-CKD demonstrated that an intake of protein below 0.8 g/kg/day was linked to a reduced risk of kidney failure and deaths from all causes. The meta-analysis further showed enhancements in biochemical markers, such as elevated serum bicarbonate levels, reduced serum phosphorus levels, and lower azotemia, in comparison with a protein intake of 0.8 g/kg/day or more [16]. Although several meta-analyses have shown evidence that lower dietary protein intake is associated with decreased proteinuria, CKD progression, and uremic complications [16,17,18,19,20], relatively large RCTs have revealed the marginal effects of a low-protein diet on the progression of kidney disease.

Modification of Diet in Renal Disease (MDRD) study 1: The MDRD compared normal (1.3 g/kg/day) and low-protein (0.58 g/kg/day) diets in 585 people (GFR 25–55 mL/min/1.73 m^2^). Poor adherence yielded actual intakes of 1.11 and 0.73 g/kg/day, respectively. Over 2.2 years, the change in GFR was not significantly different; however, the low-protein diet initially lowered GFR (likely owing to reduced hyperfiltration), then exhibited a 28% slower decline (*p* = 0.009), suggesting potential long-term renal protection. Limited use of renin–angiotensin system (RAS) inhibitors (32–44%) hindered conclusions about the efficacy of a low-protein diet versus standard therapies [21].

MDRD study 2: This study randomized 255 patients (GFR 13–24 mL/min/1.73 m^2^) to low- (0.58 g/kg/day) and very-low- (0.28 g/kg/day with keto acid-amino acid supplementation) protein diets. No significant differences in GFR values or kidney failure emerged. Actual protein intakes (0.69 and 0.46 g/kg/day) missed the targets, possibly obscuring any benefits [21]. Surprisingly, the long-term follow-up (median 10.6 years) showed an 82% higher all-cause mortality in the very-low-protein group, possibly owing to low energy intake (approximately 22 kcal/kg/day), causing protein–energy wasting [22].

RCT by Cianciaruso et al.: In this study, an 18-month study period was extended to 30 months, comparing low- (0.55 g/kg/day) and moderate- (0.80 g/kg/day) protein diets in 423 patients (estimated GFR [eGFR] <30 mL/min/1.73 m^2^, baseline mean 16 mL/min/1.73 m^2^). The actual intakes were 0.73 and 0.90 g/kg/day; 44% of patients received RAS inhibitors. After 30 months, dialysis initiation, death, and eGFR slopes showed no significant differences. Calorie adherence, verified by a dietitian, resulted in similar weight and urinary creatinine changes. The low-protein diet did not worsen nutritional status but offered no kidney or survival benefits [23].

ERIKA study by Bellizzi et al.: This was a pragmatic RCT, comparing very-low- (0.30 g/kg/day with essential amino acids and keto analogues) and low- (0.60 g/kg/day) protein diets in 223 patients (CKD stages G4 to G5). Reflecting real-world patients (30–40% with diabetes and cardiovascular issues, >75% on RAS inhibitors), poor adherence resulted in median intakes of 0.60 and 0.83 g/kg/day, respectively. Therefore, the protein intake was not actually very low. This study yielded no significant differences in kidney failure, mortality, or cardiovascular events [24].

Overall, the effects of low-protein diets and supplemented very-low-protein diets on CKD progression and mortality remain unclear, particularly with standard CKD treatments. These studies consistently showed that target protein intake levels were not achieved; however, even at the attained levels (approximately 0.8–1.0 g/kg), potential benefits were observed. Further reductions in protein intake did not yield additional benefits.

## 4. Rationale Behind Plant-Dominant Low-Protein Diets (PLADO)

### 4.1. Various Types of Plant-Based Diets

**Figure 1:**
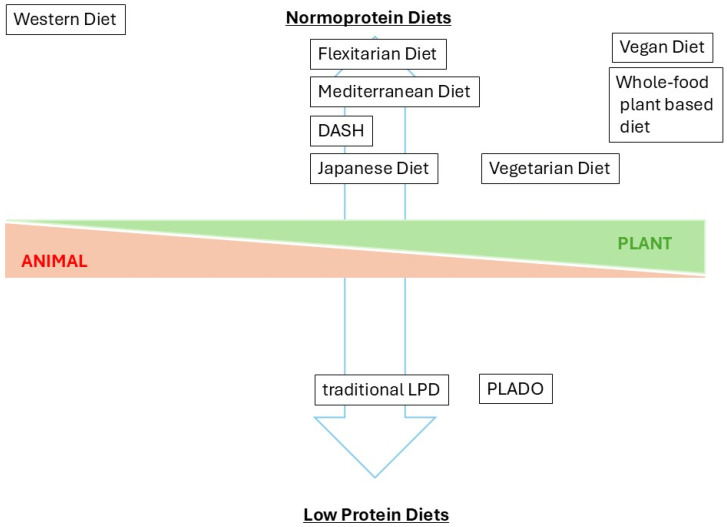
Different diets according to the content of animal and plant foods as well as that of protein. The protein content in different diets increases in the direction of the upper light blue arrow; the protein content decreases in the direction of the lower light blue arrow. DASH: dietary approaches to stop hypertension, LDP: low-protein diet, PLADO: plant-dominant low-protein diet.

The marginal results from RCTs of traditional low-protein diets have driven the search for alternatives to this approach. PLADO represents a shift from traditional low-protein diets by emphasizing the consumption of plant-based proteins over animal-based sources. Various types of plant-based dietary regimens have previously been described [15] (Figure 1), as follows:

Vegan Diet: a dietary choice that excludes all animal products;

Whole-Food Plant-Based Diet: a diet focusing on the consumption of whole, unprocessed plant foods, avoiding animal products and highly processed foods;

Vegetarian Diet: a dietary pattern that excludes meat and fish but typically includes other animal products such as dairy and eggs;

Flexitarian Diet: a flexible approach to eating that primarily emphasizes plant-based foods while allowing for the occasional consumption of meat and fish;

Mediterranean Diet: This diet emphasizes fresh, whole foods and is associated with numerous health benefits. In this dietary pattern, sugars, processed food, and healthy fats, such as olive oil, are added, but red meat is excluded;

Dietary Approaches to Stop Hypertension (DASH): DASH focuses on the high intake of nutrient-rich foods, like plant foods, in addition to reduced sodium and high calcium intake;

Japanese Diet: a diet characterized by the traditional eating pattern in Japan that emphasizes fresh, seasonal ingredients and a variety of foods like seafoods, vegetables, soy products, and fermented foods (50% plant protein) [25];

PLADO: a dietary pattern for patients with CKD that primarily emphasizes plant-based foods, intentionally restricting overall protein intake (0.6–0.8 g/kg with 50–75% of intake from plant-based sources, dietary sodium 4 g/day, and dietary energy 30–35 kcal/kg of ideal body weight/day);

PLAFOND: a dietary pattern for patients with CKD who have diabetes. The ingredients recommended for this diet are the same as those for PLADO.

The proportions of total protein intake attributable to animal and dairy protein in Western diets are as follows. The National Health and Nutrition Examination Survey (NHANES) in 2007 to 2010 indicated that the typical diet in the United States includes only 30% plant protein, with chicken and beef the two leading food categories for animal protein [26]. Similarly, approximately 35% of protein intake is derived from animal foods in the European diet [27].

### 4.2. Advantages of PLADO

**Figure 2:**
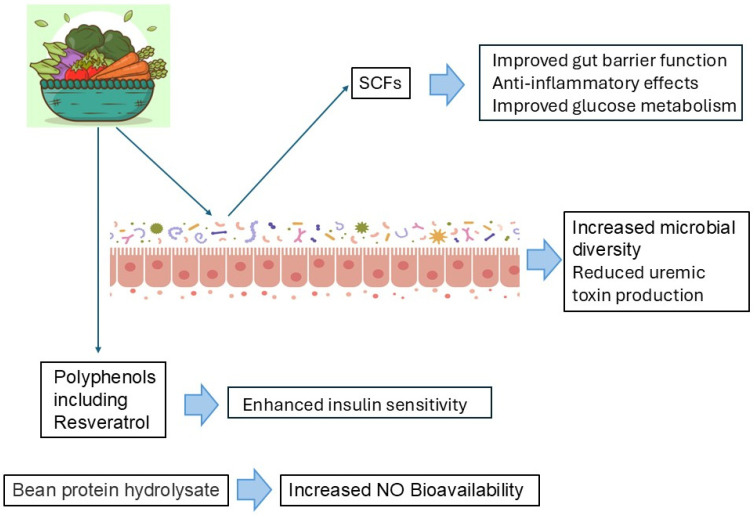
Beneficial effects and mechanisms of plant-based diets. Plant-based diets exert their beneficial effects via gut microbiota and bioactive components (polyphenols and bean protein hydrolysate). The blue arrow indicates functions, respectively. SCFAs: short-chain fatty acids, AGEs: advanced glycation end products, NO: nitric oxide.

The PLADO approach offers several advantages (Figure 2):

Improved Nutrient Density: Plant-based foods are rich in essential vitamins, minerals, fiber, and antioxidants [28], promoting overall health and potentially mitigating the adverse effects of protein restriction. This contrasts with animal-based protein sources [26], which may be higher in saturated fat, cholesterol, and phosphorus, contributing to cardiovascular disease and mineral bone disease, common complications in CKD.

Reduced Acid Load: Dietary acid load (DAL) is associated with the consumption of sulfur-containing amino acids (methionine and cystine), primarily found in animal proteins, which contribute to the production of sulfuric acid and hydrogen ions within the body [29]. Diets high in DAL induce a low-grade metabolic acidosis state, which may be associated with metabolic alterations such as insulin resistance, diabetes, and hypertension, leading to CKD progression [30,31,32]. A lower DAL can reduce strain on the kidneys and potentially improve their function.

Improved Inflammatory Profile and Oxidative Stress: Plant-based diets are associated with reduced inflammation, a key factor in CKD pathogenesis. Many plant foods contain bioactive compounds with anti-inflammatory properties, which may help to slow disease progression. In contrast to animal foods, plant-based foods are abundant in phytochemicals that possess a greater ability to neutralize free radicals [33]. Additionally, bioactive plant compounds are suggested as a non-pharmacological approach to mitigate inflammation and oxidative stress. For instance, cruciferous vegetables enhance sulforaphane intake, a nuclear factor erythroid-derived 2-related factor 2 agonist. Further, curcumin decreases nuclear factor–kappa B (NF–κB) activation, whereas beetroot, garlic, and berries provide antioxidant and anti-inflammatory benefits [34]. Furthermore, polyphenols (flavonoids, lignans, stilbenes, and phenolic acids) in tea, cocoa, fruits, and vegetable-based foods are known to serve a vital function in eliminating reactive oxygen species, which are involved in the progression of CKD [35].

Synergistical effect with CKD pharmaceutical therapy: Vegetable proteins exert a lesser impact on kidney hemodynamics than animal proteins, reducing renal loads and hyperfiltration. This impact may work together with the pharmacological effects of renin–angiotensin–aldosterone system inhibitors and sodium–glucose cotransporter type 2 inhibitors [36,37].

Increased Microbial Diversity: Plant-based diets are known to promote a more diverse gut microbiome compared with diets high in animal protein. Ismael et al. reported that an increase in bacterial diversity and richness was observed in patients with type 2 diabetes, in addition to a decrease in the Firmicutes-to-Bacteroidetes ratio, which promotes gut homeostasis [38]. Increased microbial diversity is often associated with improved overall health and resilience against various diseases [39]. Studies have shown that plant-based diets can increase the relative abundance of beneficial bacteria, such as *Bifidobacteria* and *Lactobacilli*, known for their anti-inflammatory properties and ability to produce short-chain fatty acids (SCFAs) [40,41,42].

Short-Chain Fatty Acid (SCFA) Production: SCFAs, particularly butyrate, propionate, and acetate, are produced by the fermentation of dietary fiber by gut bacteria [42]. SCFAs have various beneficial effects, including improved gut barrier function [43], anti-inflammatory effects [44,45], and improved glucose metabolism effects [46,47,48], which reduce the metabolic burden associated with CKD.

Reduced Uremic Toxin Production: A large body of evidence indicates that plant-based diets may reduce uremic toxin production, probably because of its higher fiber content [49,50]. Many protein-bound uremic toxins, including indoxyl sulfate and p-cresyl sulfate, originate from the breakdown of aromatic amino acids by the gut microbiome [42]. A plant-based diet might decrease gut-derived uremic toxins by enhancing fiber intake and altering the intestinal microbiota. High-fiber, plant-based diets increase the abundance of SCFA-producing saccharolytic bacteria while decreasing proteolytic bacteria [51], leading to lower levels of gut-derived uremic toxins [52]. Increased SCFA production fuels colonocytes and gut microbiota, enabling amino acids to be incorporated into bacterial proteins and excreted, rather than being fermented into uremic toxins [53]. In an RCT involving 40 hemodialysis people, a higher dietary fiber intake over 6 weeks resulted in a 29% decrease in free plasma levels of indoxyl sulfate [54]. Furthermore, a meta-analysis of RCTs found a significant reduction in various uremic solutes owing to dietary fiber, although data on people with NDD-CKD are limited [55]. By altering the gut microbial composition, PLADO may reduce the abundance of these toxin-producing bacteria, leading to lower levels of uremic toxins in the bloodstream.

Increased Nitric Oxide (NO) Bioavailability: NO is a crucial vasodilator produced by endothelial cells [56]. Its production is impaired in endothelial dysfunction. PLADO may enhance NO production either directly through the consumption of nitrate-rich vegetables (which are converted to nitrite and then NO in the body; [57,58]). Bean protein hydrolysate in PLADO indirectly enhances NO production by increasing NO synthase expression, probably via ACE inhibition [59]. Increased NO bioavailability leads to improved vasodilation and reduced blood pressure.

Enhanced Insulin Sensitivity: In addition to SCFAs, polyphenols in a plant-based diet also have favorable effects on insulin resistance [60]. Therefore, plant-based diets are frequently associated with improved insulin sensitivity, which can have beneficial effects on both glucose and protein metabolism.

## 5. Evidence from Clinical Studies

Several studies suggest that plant-based diets may offer benefits in reducing the risk of CKD or kidney failure [61]. A systematic review and meta-analysis with 18 cohort studies, including those with 630,108 adults that ranged from 10.4 ± 7.4 years, demonstrated that a healthy dietary pattern characterized by higher intakes of fruits, vegetables, legumes, nuts, whole grains, fish, and low-fat dairy was associated with a lower incidence of CKD (odds ratio [OR] 0.71, 0.60–0.82) and albuminuria (OR 0.77, 0.59–0.99) [62]. Another systematic review and meta-analysis involving seven studies and including 568,213 participants revealed that the DASH dietary pattern was associated with a low risk of incident CKD (pooled risk estimate: 0.71, 0.63–0.94; *p* = 0.01) [63]. Several observational studies have shown that greater adherence to more plant-based dietary patterns, such as the DASH, Mediterranean, and vegetarian diets, was associated with a lower risk of incident CKD and kidney failure [64,65,66,67]. However, research on the effects of plant-based diets on CKD progression is scarce. In a randomized controlled trial of a plant-based diet, Garneata et al. carried out an open-label, 15-month study [68] comparing a vegetarian, supplemented, very-low-protein diet (0.30 g/kg/day, supplemented with essential amino acid keto analogues) with a low-protein diet (0.60 g/kg/day, including animal proteins) among 207 people with an eGFR of <30 mL/min/1.73 m^2^. A unique aspect of this study was that only people who were expected to adhere to the diet were enrolled. Consequently, just 14% of screened people were randomized and many were excluded owing to adherence issues. Despite significant limitations and a lack of real-world applicability, in the group on a supplemented very-low-protein diet, serum bicarbonate levels were notably increased and serum phosphate, urate, and urea levels were significantly decreased. Additionally, there was a significant reduction in the primary kidney outcome rate (initiation of renal replacement therapy and a 50% reduction in eGFR reduction) in the supplemented group compared with the group on a low-protein diet (13% vs. 42%; *p* < 0.001). The number of people who needed to be treated for a year to prevent dialysis initiation was only 22.4. No significant differences in nutritional parameters were observed between the groups.

This trial strongly endorsed the effectiveness of a vegetarian, supplemented, very-low-protein diet for people with advanced CKD in lowering kidney failure risk. However, it remains unclear whether the benefits arose from strict protein restriction or the vegetarian protein sources. Importantly, this diet was not a plant-based, low-protein diet (PLADO). Future PLADO studies should explore the ideal amount of plant protein for kidney protection.

## 6. Challenges and Considerations in Implementing PLADO

Despite the potential benefits, several challenges exist in implementing PLADO.

Dietary Adherence: As has been pointed out in several clinical studies of low-protein diets, low adherence rates are common [24,68]. This is owing to, as follows: (1) the unappealing taste of protein-free alternatives, like starch-based bread and pasta, which may hinder patient adherence; (2) the higher cost of these alternatives; and (3) challenges in maintaining the diet when away from home [69]. Large dietary changes can be difficult to maintain in the long term. Traditional low-protein diet trials have shown limited success due to poor adherence and marginal benefits. In contrast, PLADO offers a potentially more sustainable alternative; its natural composition may improve adherence compared to traditional low-protein diets. Long before PLADO was introduced, Barsotti et al. proposed a special, vegan, low-protein diet, which only included natural foods of plant origin in definite proportions, to provide a supply of essential amino acids that satisfied the recommended dietary allowance [69]. This diet comprised an appropriate grain–legume mixture, supplying complementary proteins to essential amino acids and a high ratio of unsaturated to saturated fatty acids, an absence of cholesterol, and lower net acid production [69]. Nutritional counseling and support are crucial for successful implementation. Importantly, even though participants did not fully adhere to the target protein intake, potential benefits were still observed at the levels achieved.

Nutrient Deficiencies: Careful planning is necessary to avoid nutrient deficiencies in people with CKD [70], especially for vitamins B12, D, and iron, which are less abundant in plant-based foods [28]. There is another concern regarding amino acid deficiency in PLADO [71]. Plant-based foods often provide lower levels of amino acids than omnivorous diets, but they do not contain low protein in themselves. In grains, lysine is found in amounts that are lower than ideal for human needs. Similarly, the sulfur-containing amino acids methionine and cysteine are less abundant in legumes than what is optimal. Although a strictly plant-based diet focused solely on grains could potentially result in a lysine deficiency, it is also possible to acquire a considerable amount of total protein in such a diet through a high intake of low-protein foods like fruits and vegetables [72]. To improve the nutritional value of plant-based diets, Zarantonello et al. proposed the following strategy [71]:

Daily intake of legumes;

Use of nuts and oilseeds;

Consumption of primarily whole grains;

Consumption of pseudo grains;

Eating plenty of vegetables.

Potassium Management: Historically, people with CKD have been advised to limit dietary potassium to prevent hyperkalemia, which can lead to severe arrhythmias and even death. However, such restrictions, which often reduce healthy plant-based food intake, may prevent individuals from experiencing the potential benefits of high potassium consumption for declining kidney function, kidney failure, and cardiovascular risk events [73]. It is essential to note that meat, fish, and poultry also contain high levels of potassium [74]. More importantly, potassium additives found in processed and ultra-processed foods, which are common in modern diets, are absorbed almost completely. KDIGO highlights that salt substitutes, food additives, and preservatives are important hidden sources of potassium that can greatly elevate total dietary intake [74]. Furthermore, the bioavailability of potassium varies depending on the food sources. Approximately 50% to 60% of the potassium in fruits and vegetables is absorbed in the gastrointestinal tract, compared with roughly 80% in animal foods [61].

Furthermore, evidence regarding the link between dietary potassium intake and serum potassium levels in people with CKD is limited and ambiguous; many observational studies aimed at correlating serum potassium with estimated potassium from dietary records have found no significant relationships [75]. Only one recent study reported a weak association between dietary potassium intake (assessed using multiple 24 h urine samples) and serum potassium levels [76].

However careful monitoring and selection of plant-based foods are needed to manage potassium and phosphorus intake, particularly in advanced CKD. If hyperkalemia occurs, new potassium-binding agents (patiromer and sodium zirconium cyclosilicate) should be considered rather than discontinuing the healthy plant-based diet [77]. Based on recent research, Dr. St-Jules suggests that, unlike low-potassium diets, plant-rich diets address the underlying issues of potassium distribution and excretion that contribute to chronic hyperkalemia in CKD people [78].

Energy Requirement for People with CKD: The 30–35 kcal/kg recommendation in PLADO is questionable, as current guidelines generally recommend lower caloric intakes, reflecting increased global obesity and reduced activity levels compared to the populations of earlier studies [79]. Personalized approaches and individual counseling are essential for effective calorie restriction for people with CKD, especially for older people.

Avoidance of protein restriction diets: Some groups of patients, like children with CKD or undernourished subjects, should avoid protein restriction of any kind.

**Figure 3:**
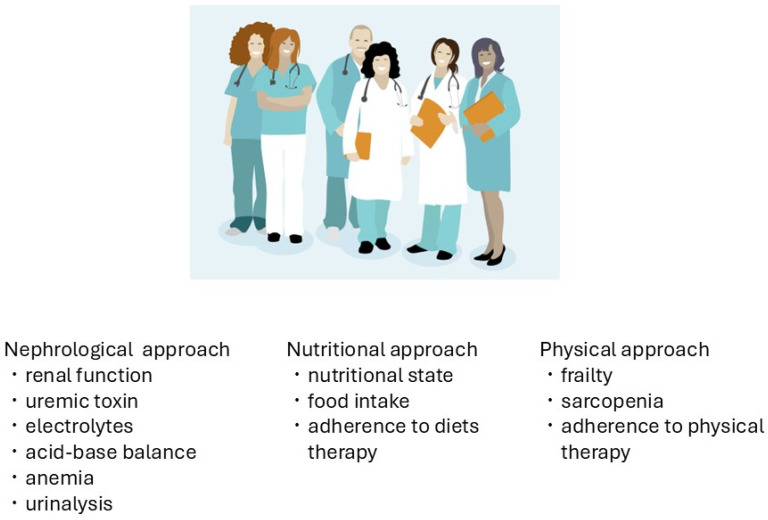
A medical team comprising nephrologists, dietitians, and exercise instructors manage patients with CKD on PLADO diet therapy using different approaches. PLADO: plant-dominant low-protein diet.

Individualized Approach by a Multidisciplinary Team: PLADO should be tailored to individual patient needs, considering factors such as the disease stage, comorbidities, dietary preferences, and cultural background (Figure 3). For this reason, supervision by a multidisciplinary team is recommended, comprising nephrologists, specialty-trained dietitians, and other medical professionals such as exercise instructors. The patient’s nutritional and physical states should be under close observation because frailty or sarcopenia may occur in people who are prescribed PLADO and are poorly adherent, especially older people [80].

## 7. Conclusions

This review has explored the current evidence surrounding PLADO in the management of NDD-CKD. While traditional low-protein diets, like those recommended in the KDOQI and KDIGO guidelines, have shown mixed results in clinical trials, often hampered by poor adherence and marginal benefits, PLADO offers a potentially more palatable and sustainable approach. The inherent advantages of PLADO, including improved nutrient density, reduced dietary acid load, anti-inflammatory effects, and modulation of the gut microbiome, suggest a multifaceted mechanism for slowing CKD progression and improving patient outcomes. The emphasis on plant-based protein sources addresses several key challenges associated with traditional low-protein diets such as higher nutrient density and reduced uremic toxin production.

However, several challenges remain. Ensuring dietary adherence remains a significant hurdle, requiring comprehensive nutritional counseling and support. Careful attention must be paid to prevent potential nutrient deficiencies, especially deficiencies in vitamins B12 and D, and iron, which require careful dietary planning and potential supplementation. While concerns exist regarding potassium management, recent research suggests that plant-rich diets may address underlying issues of potassium distribution and excretion, making strict potassium restriction unnecessary in many cases. Furthermore, the recommended caloric intake requires individualization; a multidisciplinary approach involving nephrologists, dietitians, and other healthcare professionals is crucial for successful implementation and monitoring.

The existing evidence, while promising, is still limited. Large, well-designed, randomized controlled trials are needed to definitively establish the efficacy of PLADO compared to traditional low-protein diets and standard CKD management. These trials should focus on long-term outcomes, adherence rates, and the impact of PLADO on various CKD complications. Future research should also investigate the optimal composition of PLADO, including the ideal ratio of plant-based protein sources and the role of specific bioactive compounds in mitigating CKD progression. Ultimately, a personalized approach, tailored to individual patient needs and preferences, is crucial to maximizing the potential benefits of PLADO in improving the lives of individuals with NDD-CKD.
